# Current Status of Biomarkers for Prostate Cancer

**DOI:** 10.3390/ijms140611034

**Published:** 2013-05-24

**Authors:** Vicki M. Velonas, Henry H. Woo, Cristobal G. dos Remedios, Stephen J. Assinder

**Affiliations:** 1 Bosch Institute, the University of Sydney, Sydney 2006, Australia; E-Mail: vvel9215@uni.sydney.edu.au; 2 Sydney Adventist Hospital Clinical School, the University of Sydney, Sydney 2006, Australia; E-Mail: hwoo@urologist.net.au

**Keywords:** PSA, genomics, antibody microarrays, diagnostic biomarkers, genomics, prostate cancer, proteomics

## Abstract

Prostate cancer (PCa) is a leading cause of cancer-related death of men globally. Since its introduction, there has been intense debate as to the effectiveness of the prostate specific antigen (PSA) test as a screening tool for PCa. It is now evident that the PSA test produces unacceptably high rates of false positive results and is not prognostic. Here we review the current status of molecular biomarkers that promise to be prognostic and that might inform individual patient management. It highlights current efforts to identify biomarkers obtained by minimally invasive methods and discusses current knowledge with regard to gene fusions, mRNA and microRNAs, immunology, and cancer-associated microparticles.

## 1. Introduction

Prostate cancer (PCa) is the second most common cause of male cancer-related deaths and the most common male non-cutaneous malignancy in the Western world [1]. Recent statistics from the Prostate Cancer Foundation of Australia [2] demonstrate that just as many men die each year from PCa as women die from breast cancer. In Australia alone 120,000 men live with PCa, 20,000 new cases are diagnosed per year and approximately 3,300 will die each year [2]. Globally, this equates to a PCa related death every four minutes. The rate of diagnosis dramatically increased over the past decades in part due to an ageing population, increased awareness of PCa, and the introduction of the prostate specific antigen (PSA) test [3].

Current diagnosis and “informed” treatment decisions for PCa involve digital rectal examination (DRE), PSA and subsequent biopsies for histopathological staging [4]. However, each procedure has its shortcomings and, in practice, has led to the over-treatment of low-risk patients [5], unnecessary biopsies and non-essential radical prostatectomies [6,7]. Current emerging biomarkers aim to enable the determination of an appropriate treatment strategy for individual patients, detect advanced disease at an earlier stage, and predict metastatic cancer and re-occurring disease following prostatectomy.

There is some lack of agreement on the characteristics of clinically significant or insignificant PCa. This has led to difficulties and variations among urologists when identifying patients for further treatment. By definition, clinically insignificant PCa does not contribute to PCa mortality. The main problem that faces us today is over diagnosis and treatment of patients with this form of disease (indolent PCa).

The National Cancer Institute defines a biomarker as “a biological molecule found in the blood, other body fluids, or tissues that is a sign of a normal or abnormal process or of a condition or disease” [8]. The ideal biomarker should screen for the disease and its progression, identify high-risk individuals, predict recurrence, and monitor response to treatments. It should be economical, consistent, non-invasive, easily accessible, and quickly quantifiable. This review discusses the shortcomings of the PSA test with regard to these ideals, and summarizes the most promising emerging biomarkers for PCa and the need to develop a test for PCa that can distinguish between healthy patients, benign prostatic hyperplasia (BPH), clinically insignificant cancer (indolent PCa) and clinically significant PCa (re-occurring metastatic, and castration resistant prostate cancer (CRPC)).

## 2. Prostatic Acid Phosphatase and Prostate Specific Antigen Tests

Prostatic acid phosphatase (PAP) is a glycoprotein dimer produced predominately by the prostate and was initially used as a serum biomarker for the detection of metastatic PCa [9]. Unfortunately, PAP has a low sensitivity for detecting localized disease [10] and was replaced as a test following the discovery [11] and development of the prostate specific antigen (PSA) test. PSA is a 33 kDa serine protease (kallikrein-3) that is secreted by the epithelial cells of the prostate. In normal prostate, PSA is secreted from the prostatic epithelium into the secretory ducts to contribute to the seminal fluid. However, in PCa, disruption of the basal-cell layer allows PSA to “leak” into the circulation resulting in elevated serum levels of PSA. Whilst PSA is prostate enriched it is not necessarily indicative of disease [12]. As such, levels of serum PSA may be raised by non-cancer related BPH, prostatitis, diet alterations, medications and environment [13]. Furthermore, PSA does not distinguish between stages of PCa and, significantly, does not identify metastatic PCa with the sensitivity and specificity required to make accurate therapeutic decisions [14]. Since the implementation of PSA screening among the ageing population, it has reduced the average age of PCa diagnosis from 70 to 71 years of age to 67 years of age [15]. The widespread use of PSA as a screening tool has been partly responsible for the rapid increase in PCa diagnoses in the past two decades. Although mortality associated with PCa has decreased over the years, it is uncertain whether it is due to the introduction of PSA screening or to the advances and efficacy of current PCa treatments [16].

Unfortunately, the PSA test can result in false positives. When a PSA threshold of 4 ng/mL or above is regarded as an indicator for prostate biopsy it misses between 20% and 40% of cancers [17,18]. It also falsely identifies indolent PCa causing 40%–50% of cases to be unnecessarily treated [19]. Lowering the PSA threshold has been suggested as an alternative to satisfy the current problems of the PSA test. However, when the PSA threshold is decreased there is an increased risk of identifying and unnecessarily treating indolent disease. In addition a PSA > 4 ng/mL can commonly be caused by BPH, prostatitis [20] and rarely by other human malignancies [21].

A recent study [22] showed that patients diagnosed by PSA who underwent radical prostatectomy did not have a significant reduction in all-cause or PCa mortality as compared with those diagnosed by PSA, but who opted for active surveillance over a 12-year follow-up. Although this study presents some very interesting results, the fact that only 15% of men agreed to participate may have created statistical bias. Additionally, 12 years may be insufficient to assess PCa mortality. Furthermore, it suggested that radical prostatectomy might reduce mortality for men with a PSA > 10 ng/mL with higher-risk tumors. The risks and benefits of active surveillance need to be evaluated more stringently using a larger population and longer follow-up in order to establish a true opinion on the advantages of this approach.

Two large multi-center clinical trials [23,24] has cast further doubt on the effectiveness of PSA screening. The European Randomized Study for Screening of Prostate Cancer (ERSPC) and the US-based Prostate, Lung, Colorectal and Ovarian (PLCO) Cancer Screening Trial focused on the mortality associated with PCa. The ERSPC trial concluded that in order to prevent one death from PCa, 1410 men needed to be screened and 48 treated. They also suggested that the only subjects that benefited from PSA screening were subjects in the 55–69 age group [5,25]. The PLCO trial was a smaller study (76,693 men from 10 centers in the United States) and concluded that 50% of men were over-diagnosed by PSA screening with no reduction in mortality over a 7-year period due to the slow growing nature of PCa [26]. It should be noted that this study included men who would not otherwise have been screened, and that follow-up at 7-years is insufficient to draw conclusions on mortality due to the slow growing nature of PCa.

Radical prostatectomy has a 10-year survival rate of approximately 77% [27]. The risk of death for modern radical prostatectomy is minimal but the procedure itself carries significant risk of urinary incontinence and erectile dysfunction in the short term, which is resolved over the long-term [28]. These are unacceptable risks if there is no benefit, as alluded to above. Furthermore, if a patient has an elevated PSA and abnormal prostate on DRE then biopsy, currently regarded as the only “certain” way of diagnosing PCa [29], is required. It follows that the unacceptably high false positive rates of the PSA test has led to unnecessary biopsy investigations. Biopsies themselves carry significant morbid side effects such as risk of subsequent erectile dysfunction, serious infections and urinary incontinence [30].

These issues have highlighted the need not only for a diagnostic biomarker but also for a prognostic marker for PCa. Early detection of symptomless PCa does not seem to save lives due to the very slow growing nature of PCa [31]. Those studies commonly cited as a rejection of PSA are in essence an assessment of the whole system of PCa diagnosis, including PSA, DRE and biopsy. Because of the low impact that early detection has had on saving lives, the new focus for a diagnostic test should be earlier detection of advanced disease, prediction of metastatic disease, distinguishing organ-confined versus extra-capsular invasion and recruitment to active surveillance and also more accurately inform clinical decision-making to avoid unnecessary treatments.

### Refinement of the PSA Test

Developments have refined the PSA test to increase its diagnostic accuracy, including measurement of different molecular PSA forms and rate of PSA increase. Total PSA (tPSA) refers to the sum of free PSA (unbound) and bound PSA (complexed predominantly to α-1-antichymotrypsin).

The rate of tPSA increase, as defined by total PSA velocity (tPSAV) [32], has received much interest both diagnostically and prognostically [33,34]. Prior to any diagnosis (including biopsy), or treatment, there appears little increased value of tPSAV above that of a single tPSA measure [35]. However, tPSAV has some diagnostic value with time as correlated in a study with repeat biopsies [36] and has value in informing decisions following diagnosis and treatment. Total PSA velocity is also useful if a patient has a history of repeat PSA measures where an increase above a patient’s average value is most likely indicative of a prostate disorder.

The percentage free PSA test is approved for use in men that return a tPSA of between 4 and 10 ng/mL to help discriminate between the presence of PCa and BPH [37]. It is better than tPSA alone as a predictor for biopsy [38,39]. Whilst percentage free PSA enhances the diagnostic performance over total PSA, it still produces high false negatives [40] hence refining the tPSA still fails to fulfill the necessary requirements of an appropriate biomarker.

Recently Beckman Coulter (Coulter ACCESS^®^ immunoassay system) introduced a measure of prostate health index (phi). This value is calculated from a combination of tPSA, free PSA and a measure of a truncated PSA isoform [−2]proPSA. A current systematic review and meta analysis demonstrates that %[−2]proPSA has greater accuracy than tPSA or fPSA in detecting PCa detection in men that return a tPSA of between 2 and 10 ng/mL and that [−2]proPSA and its derivative phi might predict PCa aggressiveness [41].

## 3. Biological Sampling and Tolerability

**Figure 1:**
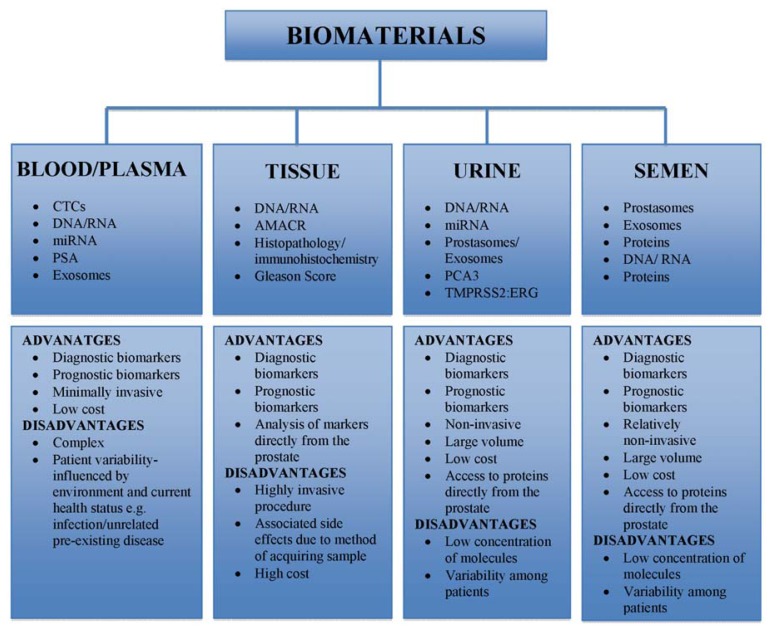
Biomaterials currently available for the identification of prostate cancer biomarkers. Common biological specimens for PCa research include blood, urine, semen and prostate tissue. Each biological sample has associated advantages and disadvantages that may affect clinical validation of biomarkers and adoption for routine testing. Human plasma contains the largest amount of human proteins that could serve as potential markers for PCa diagnosis and prognosis. Urine has become a popular source for proteomic biomarker discovery and analysis due to its non-invasive nature. It contains a vast array of markers that could distinguish between healthy BPH and malignant PCa. Semen is a relatively non-invasive material for analyzing prostate biomarkers. Proteins directly from the prostate are easily accessed; however, there is a compositional variability among patients that poses an issue. Finally, prostate tissue, although a rich source of potential PCa biomarkers, is the most invasive of sampling sites. Abbreviations: CTCs, circulating tumor cells; miRNA, micro RNA; AMACR; alpha-methylacyl-CoA racemase.

As discussed, PCa is primarily diagnosed by pathological analysis of prostate needle biopsies. Biopsies are normally collected by ultrasound-directed transrectal sampling. In addition to being invasive, biopsies also carry other significant risks, such as subsequent infection. Two recent large cohort studies [42,43] have demonstrated that there is significant hospitalization rates due to infection following biopsy, with a 3–4 fold increase above normal [44]. As highlighted by Loeb and colleagues [45], this is of concern with the increasing spectrum of antimicrobial resistant microorganisms worldwide. These increases in post-biopsy infection occurred despite the use of peri-procedural antimicrobial prophylaxis as recommended by the American Urological Association [46]. Prostate needle biopsy does not then meet the criteria required for an effective biomarker or diagnostic test. It is obviously invasive, has low tolerability and carries significant morbid risk with only samples limited to portions of the gland [46]. Hence, there is a concerted effort to identify and develop tests of highly specific biomarkers of PCa that are present in minimally invasive blood and urine samples (see Figure 1) which would be better tolerated by patients.

## 4. Current and Emerging Biomarkers

Proteomics and genomic technologies have significantly enhanced the discovery process of potential clinical biomarkers (Tables 1 and 2). Identifying biomarkers and understanding the key cancer-related pathways are essential for the development of new and improved diagnostic and predictive tools. The development of tumor-specific biomarkers has been a challenge and proteomics has contributed significantly to the identification of serum biomarkers for PCa [47].

Despite technological advances [48], serum proteomics still struggle to deal with the challenges presented by the wide range of protein concentrations, difficulty in finding low-abundance proteins due to the masking effects of high-abundance proteins [49], high levels of salts and other interfering compounds, extreme variations among individuals and lack of reproducibility which have diverted our attention to other possible biomarkers.

### 4.1. Genetic Markers of Prostate Cancer

Genomic analysis is widely used for studying disease biomarkers. Germ line genetic markers do not fluctuate over time and are available for assay at any age [112]. More than 40 PCa-susceptibility loci [113] were identified in the Genome-Wide Association Study, accounting for approximately 25% of the familial risk of PCa. This was recently expanded by the International Practical Consortium to 70 PCa susceptible loci accounting for approximately 30% of familial risk [114]. Therefore, the identification of key molecular elements in this heterogeneous cancer could lead to class-specific therapies.

Such genome wide analysis has the ability to stratify those most at risk. The report of Eeles *et al.* [114] has the ability to discriminate those top 1% of the population who have an almost 5-fold greater risk of developing the disease. Such analysis does not however distinguish the likely aggressiveness of the ensuing disease. Such markers that have great promise in achieving this are those of *BRCA1/2*. Several reports have identified an association with *BRCA2* mutations and an aggressive tumor with poor overall survival [71]. A recent large cohort study of both *BRCA1* and *BRCA2* confirms the prognostic ability of *BRCA2* mutations, but is inconclusive with regard to *BRCA1* [72].

Prostate cancer antigen 3 (*PCA3*) is a biomarker currently commercially available as a diagnostic test marketed by Gen-Probe. This non-coding RNA is only expressed in the prostate, and can be detected in urine and prostatic fluid. Whilst this does not require the collection of blood, it is considered more invasive than blood-based tests, as it requires digital massage of the prostate prior to urine collection. It is over expressed in 95% of biopsies from PCa patients compared to healthy or BPH patients with a high specificity [115]. A PCA3 score of >35 units in urine has an average sensitivity of 66% and specificity of 76% for the diagnosis of PCa compared to serum PSA (specificity of 47% and 65% sensitivity) [116].

*TMPRSS2:ERG* is the most frequent gene fusion present in PCa, accounting for approximately 90% [117] of gene fusions. The *TMPRSS2:ERG* fusion has a greater than 90% specificity and 94% positive predictive value for PCa [118]. Although a clinical diagnostic test is still not available, this marker holds great promise. Combining PCA3 over-expression, *TMPRSS2:ERG* analysis and serum PSA testing is reported to improve screening effectiveness over PSA alone [119]. Unfortunately, current evidence does not support the ability of *TMPRSS2:ERG* analysis to be prognostic with equivocal findings regarding outcome [14,18,118]. For example, *TMPRSS2:ERG* the fusion has been found in patients with good prognosis [120] and with no association of incidence with Gleason score [121].

A recent study conducted by Liong *et al.* [77] proposed a new and simple way of distinguishing between PCa and control samples. This was carried out using a blood-based microarray analysis. Gene expression was further verified using qRT-PCR and together with statistical analysis, yielded a panel of seven genes (*CTAM*, *CXCR3(CD183)*, *FCRL3*, *KIAA1143*, *KLF12*, *TMEM204*, *SAMSN1*) that could distinguish between aggressive PCa and healthy patients with a high sensitivity and specificity rate (83% and 80% respectively). The significant genes identified from blood derived mRNA have previously shown to be involved in the immune response, chemotaxis and gene transcription regulation in carcinogenesis [77,122–124].

### 4.2. Circulating miRNAs in Prostate Cancer

Micro RNAs (miRNAs) are naturally-occurring, small (22bp) non-coding RNAs [125,126], regulating the expression of more than 60% of protein-coding genes [127] and are therefore potential diagnostic indicators of tumor formation and metastasis [128,129]. PCa associated microRNAs in serum allow for minimally invasive diagnostic separation of samples from tumor burdened and healthy patients. miR-21, miR-125b, miR-221 and miR-222 (Table 1) are part of the oncogenic microRNA family that are up-regulated in human aggressive PCa [130]. miR-21 is over-expressed in PCa and other tumors acting as an oncogenic regulator leading to tumor growth [129] by silencing PTEN and other tumor suppressing genes [131]. The miR-200 family has recently generated interest in PCa research due to their lowered expression in PCa. A study of a Chinese population [123] identified a panel of five miRNA markers (let7-c, let7e, miR-30c, miR-622 and miR-1285) that differentiated PCa from benign and healthy control samples. Furthermore, combining the miRNA data with the PSA test improved PCa diagnosis. miRNA studies remain a challenge because of low nucleic acid recovery, their limited availability and data validation using independent methods [124].

### 4.3. Protein-Based Biomarkers

B7-H3, also known as CD276, is a co-stimulatory molecule that may act as an antigen-specific inhibitor of T-cell mediated anti-tumoral immunity in human cancers [13,50]. B7-H3 expression seems to worsen the prognosis of PCa malignancies, as it is observed in high pathological stage PCa [50,51]. The correlation between increased expression of this molecule with PCa stage may be useful in understanding the interaction of the immune system with prostate carcinoma and may better inform possible immune therapy intervention [13,52].

Immunohistological markers of PCa are also important in distinguishing between prostate tumor stages during biopsy analysis. Several molecules have been proposed, the most widely used marker being aplha-methylacyl-CoA Racemase (AMACR). It is expressed in 80%–100% of prostate adenocarcinomas [132] and detected in blood and urine with a high sensitivity and specificity [133–135]. AMACR also correlates with PCa metastasis and biochemical recurrence when levels are lowered [136] and its inhibitors have the potential to provide a novel treatment for castrate resistant prostate cancer (CRPC). Inter-assay variation of AMACR questions its use as a biomarker [70]. This is likely due to the presence of several variants that are better able to discriminate between normal as CaP tissues than total AMACR [137]. However, the biology must be well understood before its definite therapeutic potential can be realized [138]. Although this molecule seems to hold great promise as a diagnostic marker, it is not solely specific to PCa and thus is most useful as a prostate biopsy marker in a pathology setting [134,139].

Early prostate cancer antigen (EPCA) is a nuclear matrix protein that has received much attention culminating in the findings regarding EPCA-2 reported by Leman and colleagues being retracted. Work of others has demonstrated that it is expressed in both prostate adenocarcinoma and BPH [58]. More recent studies have shown a marked increase of EPCA in PCa [82,83] showing a correlation with tumor progression and poor post-operative prognosis [56,57]. Further investigation into this molecule is required in order to make a definite conclusion on its effectiveness as a PCa biomarker.

The multi gene calcium binding protein family, more commonly known as the S100 protein family are expressed in various solid tumors and detection may be useful for diagnosis, monitoring and possible therapeutic targets [96,98]. They are involved in protein phosphorylation, enzyme activity, calcium homeostasis, and regulation of transcription factors, macrophage activators and modulators of cell proliferation [94]. S100A2, S100A4, S100A8, S100A9 and S100A11 are specific proteins from this family associated with PCa recurrence and advanced pathological stage [64,95,98].

Human kallikrein 2 (KLK2), a possible serum marker for PCa may play a role in cancer progression and metastasis [140]. KLK2 is a secreted trypsin like protease, localized to prostatic epithelium that shares the exact 80% amino acid sequence with PSA and possibly activates and regulates PSA [141]. Studies have shown that when KLK2 and PSA are used in conjunction, PCa diagnosis is improved specifically with respect to extra-capsular extension and tumor volume [67,142]. The prognostic potential of KLK2 is still under investigation; however, it may serve as an additional biomarker to complement PSA as it also has the potential to predict biochemical recurrence in men with PSA levels less than 10 μg/L [16,143].

Recent development of a urine test based on the detection of Engrailed-2 has been utilized as a new tool for diagnosing PCa. Some studies have demonstrated it to be more reliable than PSA and DRE in detecting PCa. Morgan *et al.* [95] showed EN2 had a sensitivity of 66% and a specificity of 88% using PCa cell lines and PCa tissue, with DRE not required, proving to be a non-invasive method of diagnosis [144]. Patients with PCa generally have elevated levels of EN2 expression compared to normal prostate cells [145]. EN2 also has a strong correlation with tumor volume [146], despite it is still to be determined if EN2 can discriminate between aggressive and early stage tumors. The diagnostic and predictive value of this marker needs to be further evaluated.

Finally, an olfactory receptor known as the prostate-specific G-protein-coupled receptor (PSGR) has been shown to be specifically expressed in prostate epithelial cells [147]. Its expression is increased in PCa [81], suggesting that PSGR may play an important role in early PCa development and progression. PSGR activates major intracellular signaling cascades involved in cell survival causing an inhibition in PCa cell proliferation [82]. Their current role in tumor progression remains unknown, however there is promise that these olfactory receptors might form a new subset of potential biomarkers for the detection of PCa.

### 4.4. Immunological Biomarkers

Cancers are known to activate the cellular immune system, including the mounting of an autoimmune response to antigens presented by the tumor [62]. This is due to, for example, overexpression, as in the case of AMACR [135]. Recent developments have targeted this autoimmune response in the development of multiplex arrays to detect autoimmune signatures that outperform PSA in detecting PCa with high specificity and sensitivity [148], and discriminate between PCa and BPH [149].

Cancer activation of the immune system also induces changes in surface proteins (antigens) of leukocytes that can be detected using an extensive array of cluster of differentiation (CD) antibodies [150,151]. As previously shown with melanoma and leukemia studies, observing the pattern of cell capture enables the detection of immune cell changes which can be quantified to effectively generate an immunophenotypical signature of the cancer [150–155].

Antibody microarray technology is built on the concept that leukocytes can be immobilized by the interaction of their surface CD antigens with the anti-CD antibodies deposited as dots on a 2D surface [155] such as nitrocellulose. Anti-CD antibodies are associated with PCa, particularly CD44, CD147 and CD166 [156,157]. CD166 is significantly increased in serum from murine and human cases with CRPC and in metastatic PCa [156]. Similarly Hao *et al*. [157] showed that CD44 and CD147 are also associated with metastatic PCa that has the potential to alter the tumor microenvironment. Increased expression of CD147 is not only associated with increased risk of PSA failure and metastasis but also decreased overall survival in PCa [92]. However, the above CD markers are not PCa specific and are associated with other human diseases, diluting their diagnostic potential. In practice, better sensitivity and specificity (as determined by area under the relative operating characteristic (AUROC) curve) can be obtained by selecting a panel of CD antibodies [124] that could be specific to PCa when used in combination.

### 4.5. Microparticles

The incorporation of microparticles into diagnostic assays could enable more sensitive detection than current methods due to their origin and specificity. A recent review of membrane vesicles highlighted the importance of exosomes [158] which are released by most cancer cells [19]. These submicroscopic (30–100 nm diameter) particles [159] are secreted into the bloodstream, urine and semen [160], where they are important for cell-to-cell communication and hence are likely to play a significant role in tumorigenesis [161]. Exosomes may enable metastatic cells to avoid detection [162], evade apoptosis, and promote immuno-escape [163,164]. All exosomes express characteristic surface protein markers which enable their identification, such as CD9 [165], CD81 [166] and Alix [167]. However, they also contain specific intracellular proteins that can potentially be used to differentiate between different cancers.

Prostasomes are generated from both healthy and malignant prostate acinar cells and are secreted into seminal and prostatic fluids [168]. They range from 40 to 500 nm in diameter and are predominantly involved in the liquefaction of semen, enhancement of sperm mobility and possess immunosuppressive, antioxidant and antibacterial properties [169]. PCa patients have increased numbers of prostasomes in their semen compared to men without disease and elevated levels of these vesicles correlate with an increased Gleason score [170].

Prostasomes specifically express CD46, CD55 and CD59 [171] which play significant roles in the immune system [170]. CD59 levels are greater in prostasomes isolated from metastatic prostate cells compared to non-PCa [172]. Material can be transferred from prostasomes to cancer cells where they prevent complement-mediated cell lysis, thereby allowing the PCa cells to survive the host complement system [173,174]. Exosomes and prostasomes are derived from PCa cells and therefore carry intracellular molecules (see Table 3) that may be PCa specific, which can contribute to the discovery of novel PCa markers [132].

### 4.6. Circulating Tumor Cells

Circulating tumor cells (CTCs) may be a useful approach to monitoring disease progression, and measuring treatment effects in various malignancies. Patients with a CTC count of more than 5 CTCs/7.5 mL blood have a significantly reduced overall survival compared to patients with less than 5 CTCs/7.5 mL blood [30,175]. However, current studies are limited by the low CTC detection rate [123]. To date no significant difference has been reported between CTCs from patients with biopsy-proven localized disease and biopsy-negative disease [139]. PCa CTCs are reported to reflect those mutations present in the primary tumor e.g., TMPRSS2-ERG fusions, androgen receptor mutations, and PTEN deletion which, together with PSA, AMACR and androgen receptors, can predict the response to treatment [176,177].

The number of CTCs present in whole blood might allow for determination of cancer burden, and provide a more readily accessible source of molecular information of the primary tumor. Despite their promise and proposed function, CTC detection remains a major technical challenge [178] and their clinical relevance remains controversial. Nevertheless, this research promises to understand the biology of specific subclasses of PCa in particular, castrate resistant prostate cancer using a minimally invasive technique. CTCs have the potential to reform our understanding of cancer and enhance individual treatment (Table 3). However, the labor-intensive nature of isolating CTCs, high cost and the extremely low numbers in blood is a technical hurdle, especially in the early stages of PCa.

## 5. Conclusions

With the well-described drawbacks of the PSA test, there is a concerted effort to develop replacement-screening tools for PCa. The PSA test is currently the best biomarker for PCa recurrence and it has undoubtedly been partly responsible for the increased awareness of PCa. However, no study to date has proven that screening with PSA reduces PCa mortality. It will be a challenge to replace PSA entirely due to its minimally invasive nature and low cost but there is a pressing need to complement PSA with biomarkers that can increase the specificity and sensitivity of a screen. A panel of diagnostic and prognostic biomarkers that will work in conjunction with PSA will be ideal.

Detecting multiple analytes at the same time in one sample is ideal. Different multiplex platforms are able to achieve this, such as ELISA, mass spectrometry and antibody arrays, although each method has its advantages and disadvantages. In a typical double antibody ELISA, two markers—a primary and secondary antibody—are used to increase the accuracy, specificity and detection of the antigen of interest. However, the performance of the ELISA is dependent on the quality of the antibody and the methodology employed by the user. ELISAs also lack sensitivity when using the traditional method and are relatively time consuming. Cross reactivity of antibodies is yet another drawback of this method. The possibility of an antibody binding to more than one antigen, yielding a false-positive result is a common problem. Furthermore, the analysis of only a single antigen at a time is not ideal due to the heterogeneity of the PCa. It is highly unlikely that only one marker will sufficiently screen and accurately diagnose all patients.

The analysis of a panel of multiple biomarkers may better reflect the disease state of an individual and such multiplex assays are the focus for many groups [63,179]. An antibody array is one method that has been proposed to analyze multiple markers simultaneously from a small biological sample. This method is relatively easy to use and efficient; however, the accuracy and reproducibility of this assay must be investigated. Additionally, this platform shares some of the same disadvantages as the ELISA. Inter- and intra-laboratory variability is an issue yields different results among clinics. These issues must be addressed before it can be used for the diagnosis of PCa patients.

As with antibody arrays, mass spectrometry is able to concurrently quantify multiple protein analytes at a time from a single sample. Recent advances in mass spectrometry has acquired much attention and led to the discovery of candidate protein-based markers of disease. Generally, mass spectrometry requires small sample size, fast and easily differentiates isotypes. However, its use in a clinical setting is problematic due to time-consuming data analysis and high cost to run individual samples. Quantification by mass spectroscopy can also be an issue. Unless further advances are made where these problems are avoided, mass spectrometry is best used at a research level to identify possible markers that may form the basis of a diagnostic test for PCa.

In addition to technological issues, there are also pre-analytical methods that may prevent the discovery, replication and validation of biomarkers. Variation among sample collection, handling and storage procedures, control samples and the experience of individual operators are all elements that contribute to varying outcomes of the “same” experiment, leading to the failure of candidate biomarkers not being validated for use in clinical practice. Standard methodologies and guidelines must be developed where identical controls and statistical models are used to analyze different data sets. Finally, inconsistent patient numbers among studies and sample collection bias has contributed to the lack of success when it comes to validating potential biomarkers. Many studies limit sample collection to a particular clinic or geographical location. Blinded sample collection from a number of clinics and a statistical power analysis can contribute to further verifying the diagnostic and prognostic potential of particular candidate biomarkers. The characteristics required for a suitable replacement for PSA include high sensitivity and specificity, that a marker is quantifiable and provide rapid results at low cost using a sampling methodology that is well tolerated by patients. Any replacement test should be capable of monitoring disease progression and able to distinguish between men with clinically significant PCa from those with clinically insignificant PCa. Furthermore, in early stage disease not only should a test detect PCa but also inform an appropriate individualized treatment strategy from active surveillance to an aggressive approach of surgery or radiation/chemotherapy. Tissue samples as a biomaterial to assess biomarkers is far from ideal due to the significant sampling error associated with this technique and its highly invasive approach (Figure 1). The next generation of PCa biomarkers should ideally come from minimally invasive procedures, such as urine or blood, and a robust and reproducible methodology (Figure 1). Urine and blood collection is already part of standard pathology practice and is well tolerated by patients.

Currently, no single test can achieve the above goals and we predict that one single biomarker will not be able to fulfill the above requirements for the next PCa screening tool. Due to the heterogeneity of the disease, no one biomarker will be diagnostic and prognostic for every patient. On this basis, we surmise that the next “PSA test” will most likely be an assay employing multiple biomarkers assayed in combination using protein and gene microarrays, containing markers that are differentially expressed in PCa.

## Figures and Tables

**Table 1:** Summary of proposed genetic biomarkers for prostate cancer diagnosis and prognosis.

Marker	Product	Biological Function/Relation to PCa	Reference
*B7-H3* (CD276)	Co-stimulatory molecule	May act as antigen-specific inhibitor of T-cell-mediated anti-tumoral immunity. Increased expression worsens PCa prognosis.	[50–53]
*Ki-67*	Nuclear protein	Cell-cycle-proliferation marker. Possibly a prolific predictive marker for men with low grade, low volume PCa after radical prostatectomy. Associated with metastasis and survival outcome.	[54,55]
*EPCA*	Early Prostate Cancer Antigen Nuclear matrix protein	PCa-associated nuclear structural protein measured in serum. Expressed in prostate adenocarcinoma and benign tissue; correlation with tumor progression and poor prognosis.	[56–58]
*LAT1* (CD98)	Amino acid transporter	Primary function is to transport essential amino acids. Elevated LAT1 expression in PCa proposed as a novel independent biomarker of high-grade malignancy. LAT1 activity is considered essential for cancer cell proliferation.	[59–61]
*PCA3*	Non-coding RNA	Produced in the prostate. Overexpressed compared to non-malignant prostate tissue with a high specificity.	[2,62,63]
*PSCA*	Prostate Stem Cell Antigen, a membrane glycoprotein	Involved in the regulation of cell proliferation. Up-regulated in the majority of PCas however, exact biological function is unknown. Increased expression is associated with Gleason score, seminal vesicle invasion, and capsular invasion in PCa.	[55,64–66]
*TMPRSS2-ERG* gene fusion	Transcription factor	Secreted from prostate epithelial cells; expressed in malignant prostate tissue. Independent marker of disease progression and known marker of poor prognosis. Detected in urine; small-scale studies suggest high specificity and sensitivity.	[31,32,67–70]
*BRCA1*/*BRCA2*	Tumor suppressor	Both BRCA1 and BRCA2 are involved in maintaining genome stability as members of the ATM/ATR CHK2 DNA damage repair pathway. BRCA2 is associated with aggressive tumors and poor survival outcome. BRCA2 has prognostic ability however further experimental data is needed for BRCA1.	[71–73]
*PTEN*	Phosphatase and Tensin homologue; protein phosphatase	Tumor suppressor involved in modulating the PI3-K/AKT signaling pathway. PTEN inactivating mutations/deletion occur in many tumors and result in rapid cell growth and division. It is associated with severe tumor stage; however, PTEN is not PCa specific It is among one of the most frequent genetic inactivation’s present in PCa.	[74–76]
*PI3*K	Phosphoinositide-3-kinase; Protein kinase.	One of the most common genomic alterations in human PCa contributing to cellular transformation and cancer development. Possibly a key mechanism supporting progression toward androgen-independent PCa.	[74,75]
PCa 7 gene panel *CTAM*, *CXCR3*, *FCRL3*, *KIAA1143*, *KLF12*, *TMEM204****,****SAMSN1*	Uncharacterized, Chemokine receptor 3, Fc receptor-like 3, uncharacterized, Kruppel-like factor 12, transmembrane protein 204, and SH3 domain and nuclear localization signals 1 respectively	A panel of 7 genes derived from blood mRNA could distinguish between aggressive PCa and healthy patients with a high sensitivity (83%) and specificity (80%). Genes involved in regulating the immune response and gene transcription regulation in oncogenesis.	[77]
*MME*	Membrane metallo-endopeptidase/CD10	Inactivates several peptide hormones including glucagon, abundant in the kidney. Candidate cancer biomarker associated with PCa progression. A low level of CD10 is a possible prognostic indicator for biochemical relapse and early death as a result of lymph node metastases. Additionally may aid in personalized patient treatment/management however this marker needs to be further validated.	[78–80]
*PSGR*	Prostate Specific G protein-coupled receptor Protein-olfactory receptor	Increased PSGR expression is associated with PCa progression compared to normal tissue, possibly involved in cell proliferation. Significant PSGR alterations are observed in primary PCa cases and overexpression is associated with higher pathological stage.	[69,81]

**Table 2:** Summary of current and emerging protein biomarkers for the diagnosis and prognosis of prostate cancer.

Protein Marker	Protein type	Biological Function/Relation to PCa	Reference
Alpha-methylacyl-CoA Racemase (AMACR)	Racemase	Metabolize fatty acids in the body. Over-expressed in PCa tissue; detected with a high sensitivity and specificity in blood and urine.	[74–80]
Endoglin (CD105)	Trans membrane glycoprotein	Expressed by human vascular endothelial cells thought to play a pivotal role in endothelial cell proliferation. Elevated in prostatic fluid of men with large volume PCa.	[55,82–84]
Engrailed 2; (EN-2)	Transcription factor	Involved in early embryonic development and re-expressed by PCa cells. EN-2 detection in urine as a test for diagnosing and detecting PCa. Although further validation is required, it appears it is more reliable than PSA and elevated expression is associated with increased tumor stage.	[57–59]
Prostate-specific membrane antigen (PSMA)	Type II integral membrane glycoprotein	Overexpressed on prostate tumor cells and in the neovasculature of most solid prostate tumors, but not in the vasculature of normal tissues. May play an important role in the progression of PCa.	[55,85–87]
Caveolin-1	Integral membrane protein	Mediates aspects of cholesterol and fatty acid metabolism. Circulating levels of serum Caveolin-1 correlate with extent of PCa.	[88,89]
Interleukin-6 (IL-6)	Cytokine	Involved in hematopoiesis and mediates B cell differentiation. Clinical studies reveal increased serum IL-6 concentrations in patients are associated with advanced PCa tumor stage.	[55,90]
CD147	Membrane glycoprotein	Over-expressed in many human solid tumors. Involved in tumor invasion and angiogenesis. Increased expression of CD147 is associated with PCa progression and poor prognosis. May serve as an independent predictor of biochemical recurrence and development of PCa metastasis.	[91–93]
S100 Protein Family	Calcium-binding-protein family	Expressed in various solid tumors. Detection may be useful for diagnosis, monitoring and possible therapeutic targets. Involved in protein phosphorylation, enzyme activity, calcium homeostasis, and regulation of transcription factors, macrophage activators and modulators of cell proliferation. S100A2, S100A4, S100A8, S100A9 and S100A11 are associated with PCa recurrence and advanced pathological stage.	[94–98]
Annexin A3 (ANXA3)	Cell adhesion protein	A calcium and phospholipid binding protein, primarily found in urine. Implicated in cell differentiation, migration and immunomodulation. Increases the specificity and ability of PSA to discriminate between PCa stages.	[99–103]
TGF-Beta 1	Cytokine	Growth factor involved in the regulation of cellular proliferation, immune response and differentiation. Increased expression correlates with severe tumor grade, tumor invasion, PCa metastasis and biochemical recurrence. TGF-Beta needs to be validated before becoming a PCa biomarker.	[74,104–107]
Human Kallikrein-2 (KLK2)	Serine protease	Serine protease that is highly expressed in prostate tissue and involved regulating semen liquefaction by activating pro-KLK3 to its active form (PSA), facilitating both tumorigenisis and disease progression to the advanced stages of PCa. Studies have shown a strong correlation with PCa-specific survival however further studies with larger cohorts are needed to confirm these observations.	[68,108,109]
Beta-microseminoprotein (MSMB)	Immunoglobulin binding factor	Secreted by epithelial cells of the prostate as well as other major organs. MSMB is a member of the immunoglobulin binding family. Exact function of MSMB is unknown but may have an autocrine (inhibin-like) role. The genetic variant rs10993994 is associated with PCa risk however further investigation is required to evaluate the predictive value of this marker.	[110,111]

**Table 3:** Summary of diagnostic and prognostic potential of circulating tumor cells (CTCs) prostasomes and exosomes in prostate cancer.

Category	Summary	Reference
Circulating tumor cells (CTCs)	CTCs detected in blood have been proposed for monitoring disease progression and evaluating effectiveness of cancer therapy. They carry important information specific to tumor type and stage however low CTC detection in blood proves to be a technical hurdle. Prostate cancer derived CTCs possess those same mutations present in the primary tumor (*PTEN*, *TMPRSS2*, *AMACR*), which may provide a more readily accessible source of important prognostic information for patients. To date the high cost associated with their analysis and controversial clinical relevance has prevented their use in clinical setting.	[175,176]
Prostasomes	Prostasomes are sub-micrometer membranous vesicles, generated from normal and malignant prostate cells. They are found in blood, urine, semen and prostatic fluid. An increased abundance of prostasomes have been associated with PCa and elevated Gleason score. They carry specific markers (CD46, CD55, CD59) that play a role in the immune system. Additionally, they carry specific molecules, both intracellular and extracellular, that may be specific to PCa and aid in the discovery of new PCa biomarkers.	[170,173,174]
Exosomes	Exosomes are cell-derived vesicles isolated from blood, urine and cell lines. Exosomes are released from most cancer types and possess immunosuppressive properties thought to play a significant role in oncogenesis. All exosomes express specific markers (CD9, Alix, CD81) that enable easier detection and isolation. In addition to these common markers, exosomes express specific markers unique to PCa and the cells from which they were derived. They can potentially characterize different stages of PCa and hold prognostic potential.	[159,161]
